# Understanding deep learning in land use classification based on Sentinel-2 time series

**DOI:** 10.1038/s41598-020-74215-5

**Published:** 2020-10-14

**Authors:** Manuel Campos-Taberner, Francisco Javier García-Haro, Beatriz Martínez, Emma Izquierdo-Verdiguier, Clement Atzberger, Gustau Camps-Valls, María Amparo Gilabert

**Affiliations:** 1 Environmental Remote Sensing group (UV-ERS), Universitat de València, 46100 Burjassot, Valencia Spain; 2 Institute of Geomatics, University of Natural Resources and Life Sciences, Vienna (BOKU), Peter Jordan Str. 82, 1190 Vienna, Austria; 3 Image Processing Laboratory (IPL), Universitat de València, 46980 Paterna, Spain

**Keywords:** Environmental sciences, Environmental social sciences, Climate-change policy, Environmental economics, Agroecology, Computer science

## Abstract

The use of deep learning (DL) approaches for the analysis of remote sensing (RS) data is rapidly increasing. DL techniques have provided excellent results in applications ranging from parameter estimation to image classification and anomaly detection. Although the vast majority of studies report precision indicators, there is a lack of studies dealing with the interpretability of the predictions. This shortcoming hampers a wider adoption of DL approaches by a wider users community, as model’s decisions are not accountable. In applications that involve the management of public budgets or policy compliance, a better interpretability of predictions is strictly required. This work aims to deepen the understanding of a recurrent neural network for land use classification based on Sentinel-2 time series in the context of the European Common Agricultural Policy (CAP). This permits to address the relevance of predictors in the classification process leading to an improved understanding of the behaviour of the network. The conducted analysis demonstrates that the red and near infrared Sentinel-2 bands convey the most useful information. With respect to the temporal information, the features derived from summer acquisitions were the most influential. These results contribute to the understanding of models used for decision making in the CAP to accomplish the European Green Deal (EGD) designed in order to counteract climate change, to protect biodiversity and ecosystems, and to ensure a fair economic return for farmers.

## Introduction

The European Commision (EC) recently proposed the Resource-efficient Europe initiative^1^ under the Europe 2020 strategy for a sustainable growth via a resource-efficient, and low-carbon economy. In this aspect, the EC adopted also a new regulation for the Common Agricultural Policy (CAP) that promotes the use of remote sensing (RS) data for monitoring parcels, evaluates cross-compliance and eventually provides subsidy payments to farmers^2^. The basic payment aims at avoiding the abandonment of agricultural parcels. The green direct payment, also known as “greening”, supports farmers who undertake agricultural practices that benefit the environment and meet climate objectives. In particular they should diversify crops, maintain grasslands, and allocate 5% of arable land to areas that improve biodiversity. To put this in context, in 2018 a total of 73.7 million Euros were paid to farmers in the Valencian Community (Spain) whereof 18.8 million and 9.6 million were dedicated to the basic and greening payments, respectively. These actions are supported by land use classifications obtained from RS data. This requires on one hand a good crop identification, and on the other hand classification interpretability, which is key to provide fair assignments.

The categorisation of remotely-sensed images is usually achieved using machine learning algorithms, being deep learning (DL) the most accurate paradigm. DL has recently raised up as a discipline used in RS and Earth sciences^3^. A variety of geoscience topics dealing with extreme weather patterns^4^, climate change projections^5^, precipitation nowcasting^6^, and carbon fluxes prediction^7^ can be found in the literature. There is also a wide range of RS topics such as image fusion^8^ and registration^9^, change detection^10^, image segmentation^11^, and (drought) forecasting^12^ that involve DL methods. Nonetheless, the vast majority of RS studies dealing with DL techniques are dedicated to classification including scene identification, land use and land cover (LULC) classification, and object detection^13–18^.

RS classification approaches mainly exploit information derived from the spatial and spectral domains of a single image, and also the temporal information in the case of using image time series. DL classification algorithms that use RS data can be roughly differentiated in two major groups: techniques that design convolutional neural networks (CNNs) architectures for spatial learning, and recurrent neural networks (RNNs) for sequential learning. For a comprehensive overview, we refer the reader to excellent reviews of DL techniques and applications in RS provided by Zhang et al.^19^, Zhu et al.^20^, and Ma et al.^21^. CNNs are composed of multiple layers that are the result of performing spatial convolutions typically followed by activation units and pooling. RNNs are able to deal with sequences of data (e.g., RS time series), in such a way that the output from the previous time step is fed as input to the current step. However, RNNs suffer the vanishing gradient problem, which may lead to stop the network from further training^22^. Long short-term memory (LSTM) networks are a particular type of RNNs that mitigate the vanishing gradient problem since they employ a forget gate that varies at every time step and decides what information is retained and forgotten/erased^23^.

DL approaches usually outperform other (shallow) machine learning techniques in terms of overall accuracy (OA)^24–26^. However, the understanding of these techniques is limited^27^, and typically, the better the learning of an algorithm the more difficult its interpretation is^28^. This lack of interpretability is a major point to consider when using these algorithms. For many users it is not only important to use an algorithm that provides high accuracy but also to know how the algorithm is reaching the provided predictions^29^. The interpretability of predictions becomes a critical aspect when they are used as rationale for decision making, such as in medicine, business or in the banking/payment sector^30–32^.

Recently, some approaches have been proposed to evaluate the interpretability of deep learning models^33,34^ including methods based on model decomposition, sensitivity analysis, and feature visualisation. The relevance of network inputs can for example be obtained by the gradient-based sensitivity analysis (GBSA), which computes the prediction function squared partial derivatives with a standard gradient backpropagation^35^. The Layer-wise Relevance Propagation (LRP)^36^ propagates the prediction backward in the neural network using propagation rules until the input features are reached. Arras et al.^37^ proposed a LRP for LSTM networks that provided better results than the GBSA on a five-class prediction task. Class activation maps were used to point out the most discriminative regions used by a CNN to identify a class^38^.

**Figure 1:**
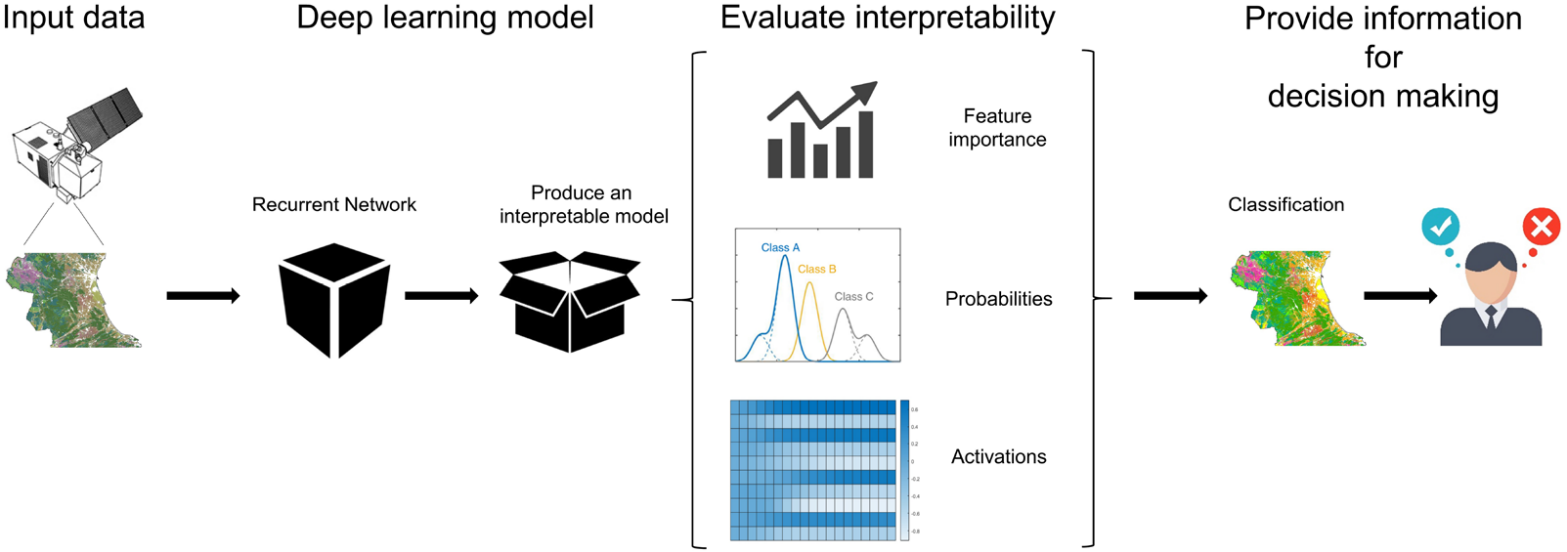
Scheme of the proposed approach for deepen understanding of a recurrent neural network for land use classification based on remote sensing data in the context of the European Common Agricultural Policy (CAP).

In the field of RS there is a lack of studies that have delved into the interpretability of DL outputs. Wolanin et al.^39^ derived regression activation maps providing information about predictions (crop yield) also retaining the correspondence with the inputs (meteorological and satellite data). Marcos et al.^40^ provided Semantically Interpretable Activation Maps (SIAM) indicating the presence of predefined attributes at different locations of an image. Pelletier et al.^41^. developed a temporal CNN applying convolutions in the temporal domain in order to quantitatively and qualitatively evaluate the contribution of network for crop mapping, as compared to RF and bi-directional RNNs with stacks of Gated Recurrent Units (GRUs). Rußwurm and Körner^42^ proposed an encoder structure with convolutional recurrent layers, and visualised internal activations over a sequence of cloudy and non-cloudy Sentinel-2 images for crop classification. It is worth mentioning that a procedure used for improving the performance of RNNs is the attention mechanism (AM). AM implements a coding-decoding model for identifying network key features^43,44^. AM has been applied in different topics such as time travel prediction^45^ or text classification^46^. In remote sensing and image processing AM has been used for improving classification in very high-resolution images^47,48^ as well as to capture the spatial and channel dependencies^49^. In this context, this work aims at evaluating the interpretability of a DL algorithm based on a 2-layer bi-directional Long Short-Term Memory network (2-BiLSTM) for land use classification over the province of València (Spain) in the framework of CAP activities (Fig. 1 shows an scheme of the process). Sentinel-2 time series during the 2017/2018 agronomic year were used as inputs for the classification. The influence of the various spectral and temporal features on the classification accuracy was assessed by means of an added-noise permutation approach in both temporal and spectral domains. The network temporal predictive behaviour was explained for every date throughout the agronomic year. In addition, different network architectures were designed and assessed, and a comparison in terms of accuracy with a set of widely used machine learning algorithms has been carried out.

## Results

**Figure 2:**
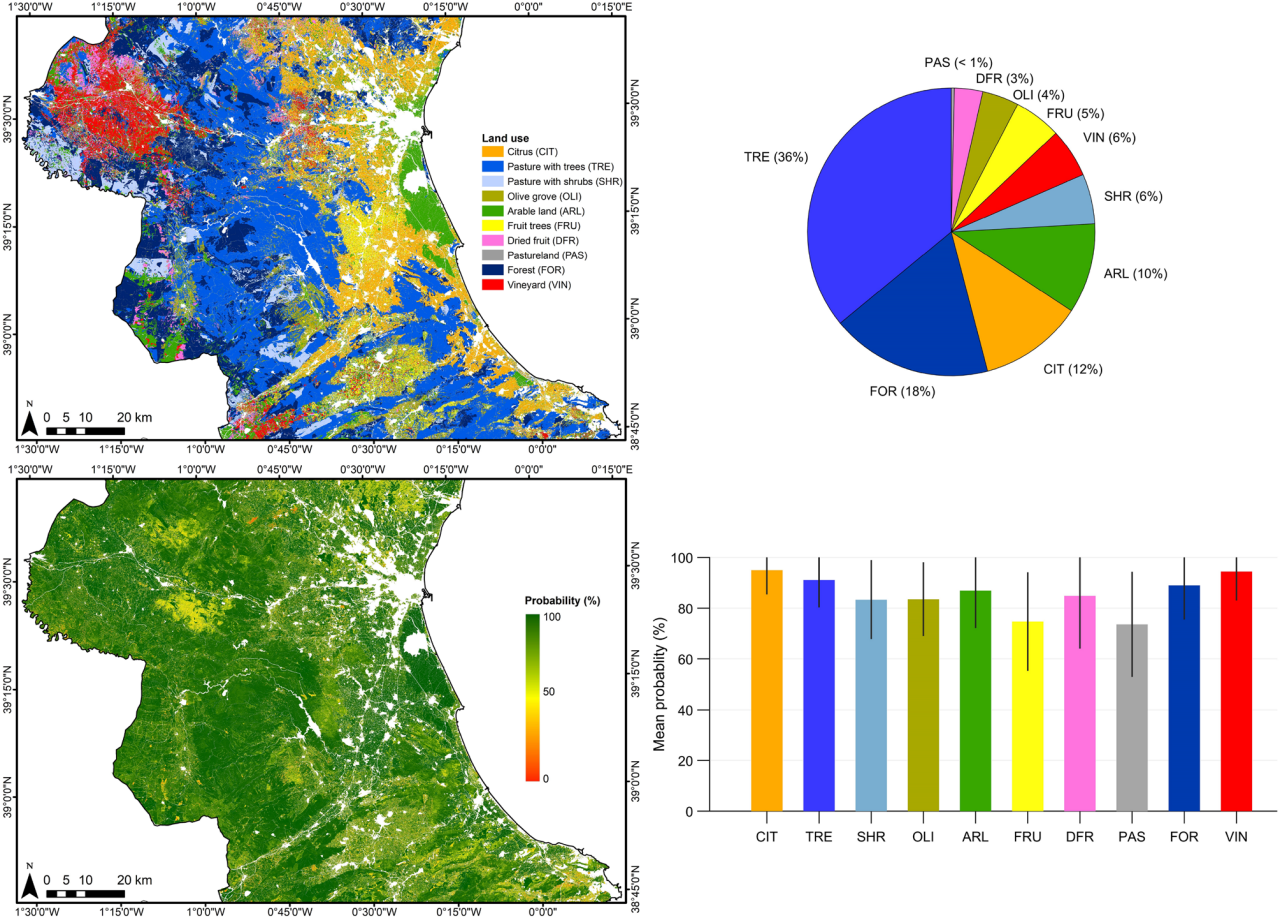
Classification map derived using the 2-BiLSTM model and the associated spatial distribution (up), and the pixel probability map and associated class mean probabilities (bottom). For the sake of visualisation, parcels belonging to rice, fallow, barley, oat, wheat, sunflower, and triticale have been grouped and displayed as arable land (ARL). Non interest areas have been masked out. Error bars indicate the standard deviation of every class probability. The maps were generated with the Arcmap v.10.5 software (https://desktop.arcgis.com/es/arcmap/).

Figure 2 shows the classification map obtained in the area using the 2-BiLSTM model. The derived classification map exhibits a spatial distribution of classes in accordance with the Land Parcel Identification System (LPIS) of Spain also known as Sistema de Información Geográfica de Parcelas Agrícolas (SIGPAC)^50^, namely: fruit trees and citrus crops in coastal zones, arable lands in the west belonging to diverse cereal crops, and rice crops in the east nearby the Albufera Natural, and natural vegetation, dried fruits, and vineyards mainly in inland zones. The 60% of the area is classified as natural vegetation (TRE, FOR, SHR, and PAS) (see Fig. 2), whereas the 24% is occupied by permanent crops (DFR, FRU, CIT, OLI), and the remaining 16% is classified as annual crops including vineyards (VIN) and arable lands (ARL) that comprises rice, fallow, barley, oat, wheat, sunflower, and triticale. Figure 2 (bottom) shows the probability map with which every pixel is classified by the 2-BiLSTM network. All classes reported a mean probability $\ge$ 83% except FRU and PAS in which the mean probability was 73%, and 72%, respectively. The vast majority of the area was classified with a high level of confidence (see greenish areas in Fig. 2, bottom). However, there exist low confidence zones (red pixels) in which the classifications should be taken carefully.

The proposed 2-BiLSMT network yielded an overall accuracy (OA) of 98.7% over the test set that was never used in the training. This accuracy outperformed the ones obtained by the rest of BiLSTM architectures as well as other classification algorithms (see Table S1 in Supplementary information). It is worth mentioning that in order to identify possible spatial bias in the classification results, different random selections of the train/test sets (preserving the 70%/30% proportion) both at pixel and parcel-based approaches were conducted, and no significant changes in performance were obtained for all the evaluated methods. The 2-BiLSTM network performed well over all classes. The confusion matrix obtained with the 2-BiLSTM (see Fig. S1 in Supplementary information) highlights the great precision of the classification algorithm over all classes. Table 1 shows the precision, recall, and F-1 score obtained for every class. The best precision was achieved over rice fields (99.9% in both user and producer accuracy) whereas the lowest one was obtained over fruit trees with a 7.4% and 8.6% of predicted, and true negative rates, respectively (see Fig. S1 in Supplementary information). The 2-BiLSTM network classified all classes with an accuracy $\ge$ 91.4% in precision, recall, and F-1 score. The 2-BiLSTM network performed also excellent over natural vegetation such as forest (FOR), and pasture with trees (TRE) classes, revealing precision, recall and F-1 score $\ge$ 99% (see Table 1). Permanent crops such as fruit trees (FRU), and citrus (CIT) were more difficult to distinguish between them (see Fig. in Supplementary information). The same applies to vineyard (VIN), olive grove (OLI), and dried fruits (DFR), in which greater confusion is reported by the confusion matrix (see Fig. S1 in Supplementary information). The discriminatory power of the classifier among classes belonging to permanent crops is slightly lower if compared with the annual crops. This is mainly due to that differences in the temporal remotely sensed signal on permanent crops is lower than the ones on annual crops in which the phenological development influences more on temporal changes in reflectance.

**Table 1:** Performance (precision, recall, and F-1 score) of every class obtained with the 2-BiLSTM network over the validation set.

Class	Precision (%)	Recall (%)	F-1 score (%)
RIC	99.9	99.9	99.9
FOR	99.7	99.8	99.7
TRE	99.4	99.5	99.4
PAS	98.9	97.5	98.2
SHR	98.8	98.9	98.8
WHE	98.5	97.6	98.0
VIN	98.2	98.5	98.3
CIT	97.9	98.4	98.1
TRI	97.9	97.6	97.7
BAR	97.7	97.4	97.5
DFR	97.0	97.8	97.4
FAL	95.8	94.6	95.2
OAT	95.4	94.4	94.9
SUN	94.4	94.0	94.2
OLI	93.9	91.1	92.5
FRU	92.6	91.4	92.0

**Figure 3:**
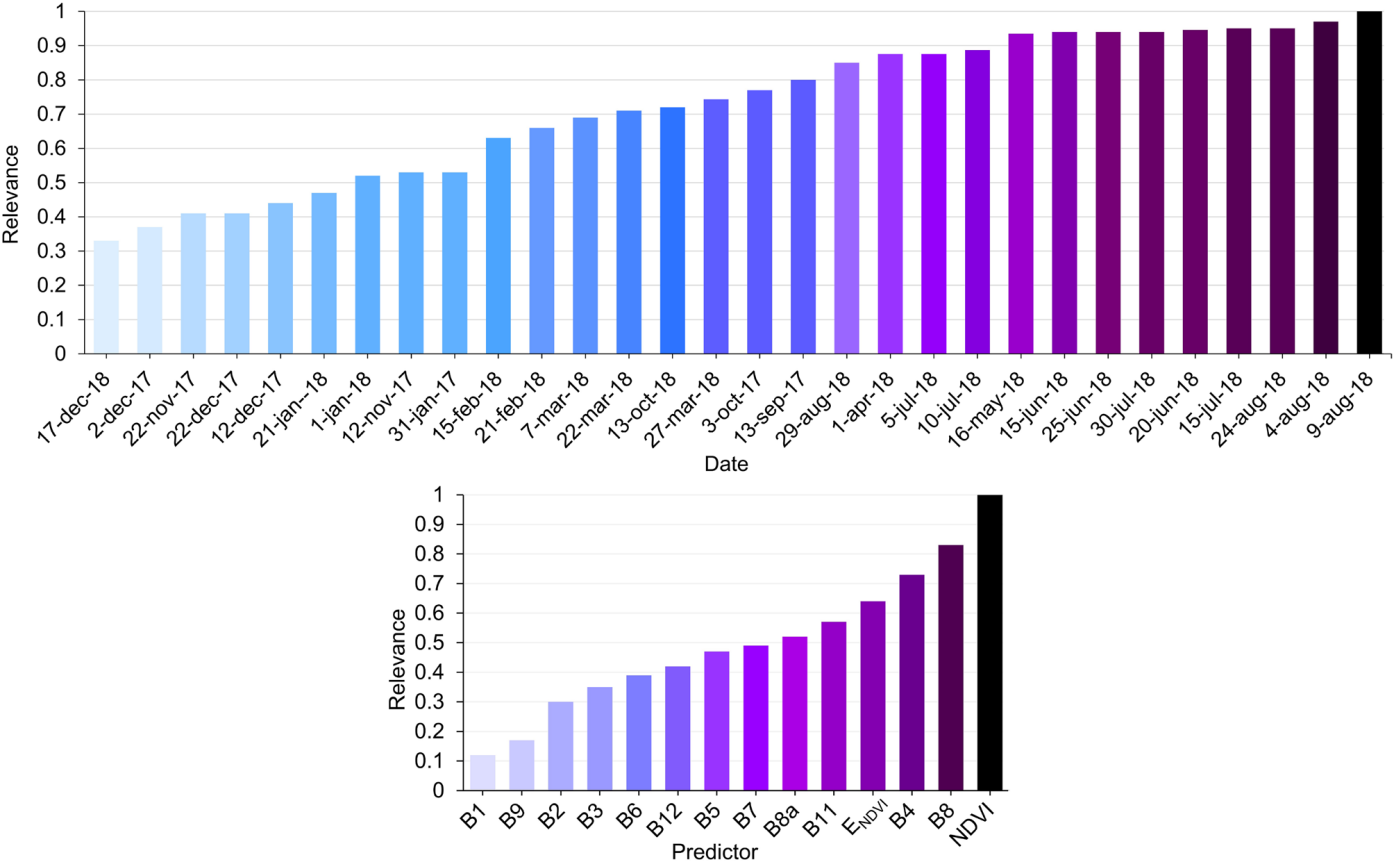
Relevance of every date (top) and predictor (bottom) in the 2-BiLSTM network.

The relevance of every Sentinel-2 date, and derived predictors in the 2-BiLSTM model is exhibited in Fig. 3. Across the temporal domain, the information provided by the Sentinel-2 image acquired on August 9th, 2018 was the most relevant, while the relevance of the image acquired on December 17th, 2018 is $\approx$ 66% lower. The NDVI temporal sequence is the most relevant predictor used by the 2-BiLSTM network. On the contrary, the Sentinel-2 aerosols band (B1) provides the least-used information by the network ($\approx$ 90% lower compared to NDVI). The relevance provided by the 2-BiLSTM model was compared with the one provided by RF classifier. The most important attributes for making the RF predictions were the NDVI of differente dates, as well as the entropy of NDVI, and the red and nir channels (see Fig. S2 in Supplementary information). This is in accordance with the results provided by the 2-BiLSTM network. However, RF seems not to be aware of the temporal information since the most relevant predictors belong to disjointed dates in terms of classes’ phenology.

**Figure 4:**
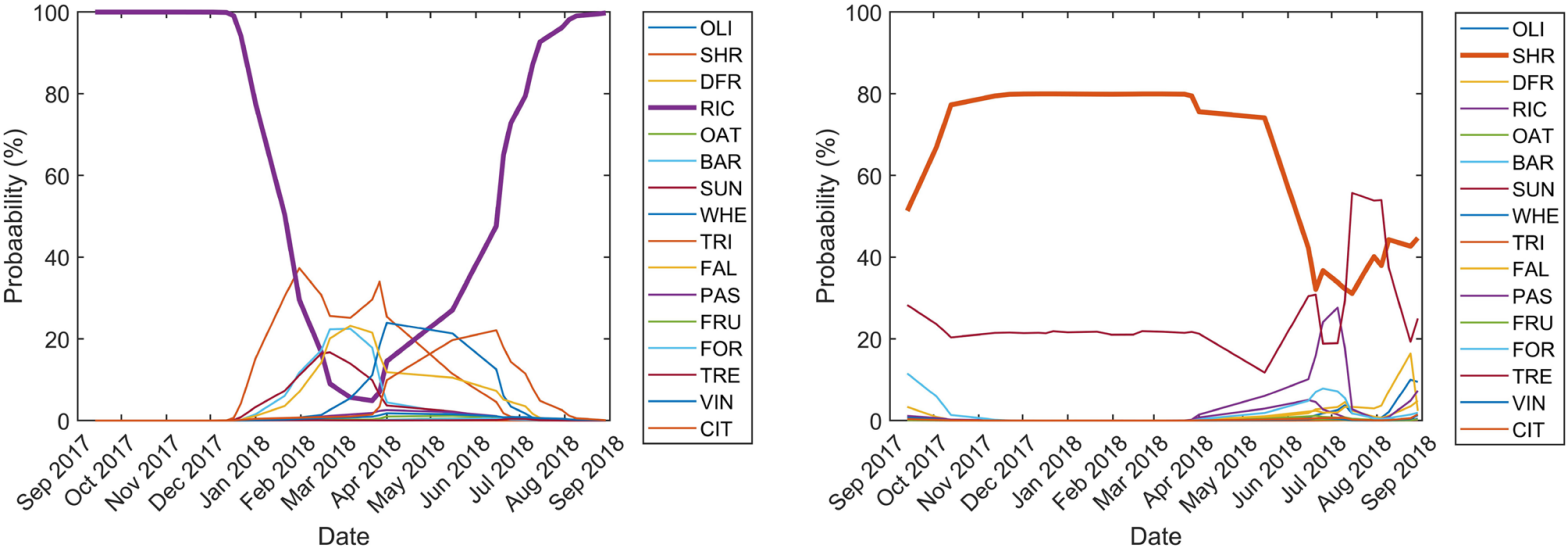
Probability evolution along time steps for representative rice (left) and pasture with shrubs (right) pixels.

The temporal predictive performance of the 2-BiLSTM network over two correctly classified pixels (one for natural vegetation, and one for crops) is shown in Fig. 4 in order to understand how the classifications are finally obtained. Over a pixel located within an homogeneous rice field (Fig. 4, left), the rice classification probability (purple line) presents the lowest values during the pre-sowing (winter–spring) period. During this time, the per class probabilities of all classes are similarly low. There is a sharp increase in the probability from the beginning of the rice cycle^51,52^ (mid-may) until the full development/maturity in august, reaching a maximum (100% probability) from the period from senescence to the end of autumn. In the case of a pixel located within a pasture with shrubs (SHR) site (Fig. 4, right), the SHR classification probability (orange line) shows high values almost over the entire period, and in particular during winter–spring when crops do not ‘interfere’ in the classification. However, even though the final classification is correctly assigned to SHR, there is a non-negligible probability assigned to the class pasture with trees (TRE) that is sometimes higher than the SHR probability. This is partly due to the spectral similarity between classes.

**Figure 5:**
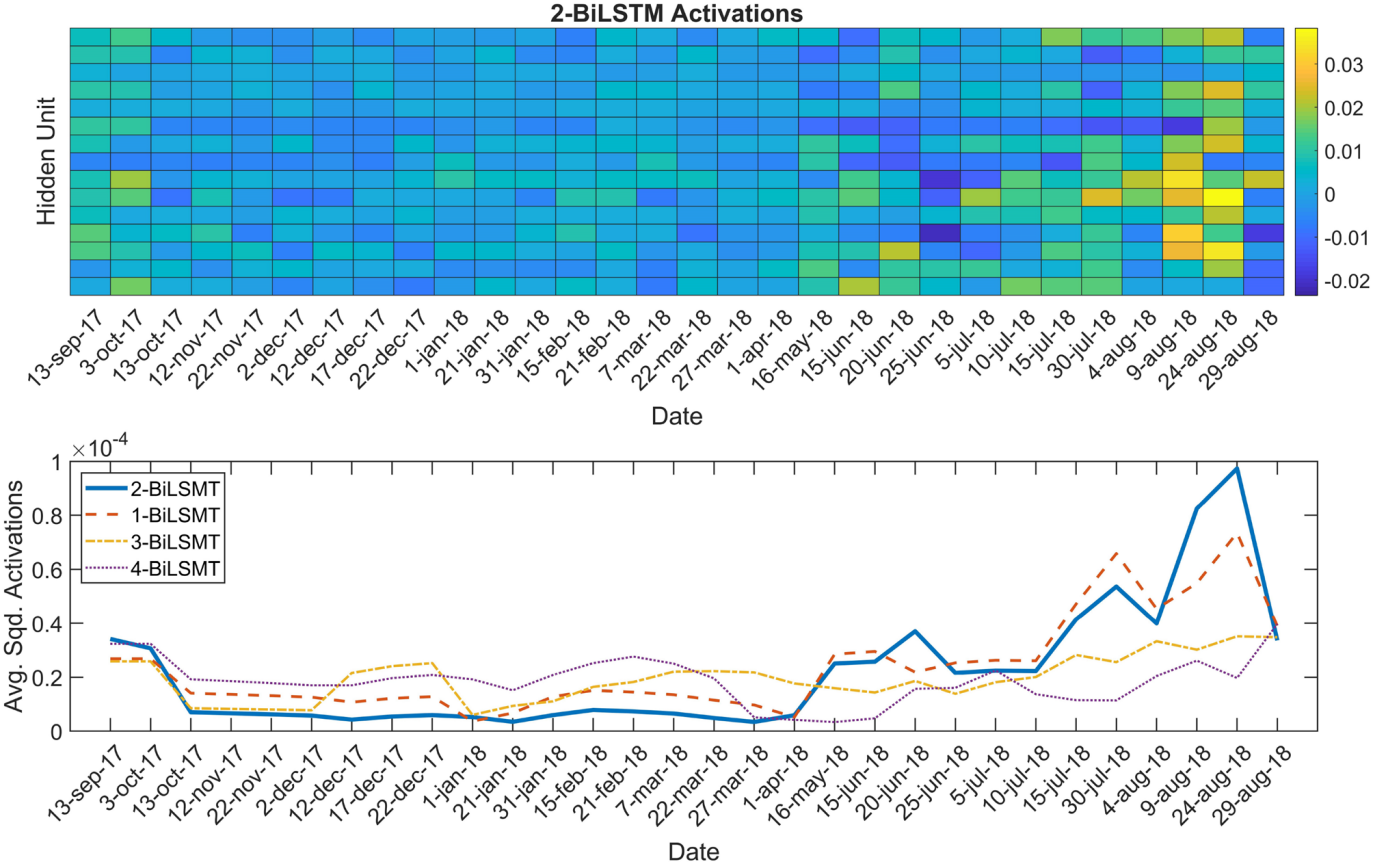
Heatmap of the 2-BiLSTM activations for every date (top), and mean squared activations in the temporal domain for the four considered BiLSTM architectures (bottom).

**Figure 6:**
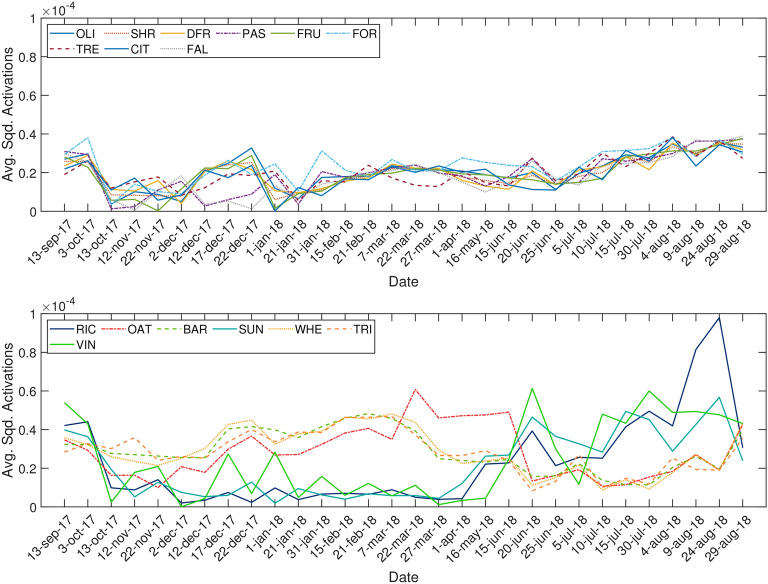
Activations observed in the 2-BiLSTM network for (top) natural vegetation and permanent crops, and (bottom) annual crops.

The 2-BiLSTM network most activated hidden units are shown in the heatmap displayed in Fig. 5 (top). The greenish and yellowish tones which pop-up, belong to the most activated hidden units, and generally correspond to summer acquisitions. To better quantify the activations along dates, Fig. 5 (bottom) shows the mean squared activations in the temporal domain of all considered BiLSTM architectures. The 2-BiLSTM and 1-BiLSTM networks present a similar behaviour with higher activations in summer. However the 3-BiLSTM, and specially the 4-BiLSTM network reveal more homogeneous activations along dates. Figure 6 (top) shows the activations of land uses belonging to natural vegetation (SHR, PAS, FOR, TRE, and FAL), and permanent crops (OLI, DFR, FRU, CIT). These classes present similar activations along time. Regarding annual crops, the activations are higher during the main phenological activity of each class (Fig. 6, bottom). For example, RIC, VIN, and SUN crops show higher activations during summer dates, whereas OAT, BAR, WHE and TRI activations are higher in winter–spring according to their phenological behaviour in the area.

## Discussion

Differentiating and identifying vegetation types with high level of confidence using RS data is possible if long enough time series of images are available^53,54^. In addition, a high temporal frequency is required to cover and characterize crop-specific phenological cycles, and to benefit from the differences established between the different classes along time. The high spatial–temporal resolution of the Sentinel-2 constellation, consisting of two identical satellites with 13 spectral bands and a combined revisit frequency of maximum 5 days, is especially well suited for identifying vegetation types and for studying vegetation dynamics.

In RS image classification, exploiting the temporal domain with RNNs is of paramount relevance. Conventional RNNs, however, present instabilities and problems during the training phase because backpropagated gradients tend to fade over time, which produces difficulties with learning long-term dependencies. LSTM networks mitigate this by incorporating a series of steps to decide which information is going to be stored (“memorized”), and which deleted (“forgotten”). Thus, the network has a certain “memory”. Furthermore, if the memory provides information on both past and future states as in the case of BiLSTMs, its use in applications where it is convenient to learn from the whole time series is of special interest.

When classifications are to be used for decision making such as the CAP, it is convenient not only to report results in terms of accuracy, but also to provide an explanation of what is internally happening in the classifier in order to subsequently interpret and explain the results. This lacking assessment is one of the main challenges DL algorithms are currently facing, since these algorithms are often seen as “black boxes” that perform high accuracy classifications without the operator being able to interpret what is happening in the algorithm. In this regard, the proposed procedure for evaluating the comprehensibility of the network revealed that the network mainly extracts information from the temporal evolution of the NDVI, the near-infrared (B8) and red (B4) bands, and the spatial information provided by $E_{NDVI}$. This aspect confirms the relevance of the Sentinel-2 near infrared, and red bands to categorise vegetation, as these two bands well address differences in leaf area index (LAI) and leaf pigmentation, respectively^52^. In a similar way, the fact that Band 11 (SWIR) also scored relatively high underlines the effectiveness of the proposed scheme, as this band is known for providing independent information related to crop water and/or protein content^55^. Several recent studies using Sentinel-2 data have highlighted the importance of this band for crop type identification^50,56^.

According to the per date relevance analysis, the network mainly uses the information from the Sentinel-2 images acquired in summer, which is consistent with the phenological activity of most of the classes identified in the study area. Likewise, it is plausible that the winter period does not offer many clues for crop type identification.

The classification results confirm that the use of two BiLSTM layers improves the OA compared to the rest of evaluated classifiers (see Table S1 in Supplementary information). The highest precision was obtained on rice crops (RIC) where practically all the pixels are correctly classified thanks to its unique crop cycle and planting pattern. This result highlights the usefulness of Sentinel-2 multitemporal data for characterizing rice as also reported in other studies^50^. Regarding the architecture of the recurring networks, the 2-BiLSTM network produced the best results. It is worth mentioning that the increase of the number of layers in a deep neural network does not necessarily lead to better classification results. In fact, the results show a clear decreasing accuracy in the case of 4-BiLSMT, which is even outperformed by the RF algorithm. This is partly because in multi-layered architectures, even though dropout layers are used, networks may tend to overfit and lose generalisation power thus decreasing accuracy. In addition, the vanishing gradient problem may also persist in architectures made by high number of layers. In the case of the 4-BiLSTM network we also found that the activations are quite similar along dates, which means there is no clearly relevant period used by the network.

The results on the temporal predictive performance of the 2-BiLSTM network reveals how the network adapts both the per class probability and the classifications along time steps. The activation of the hidden units reveal how the information is flowing through the network. Results showed that the most activated units belong to summer dates. This means that the network is giving more importance to those dates since the wealth of information is higher. This result goes in line with the results obtained in the added-noise permutation results.

## Conclusions

The use of satellite observations for land use identification and monitoring is one of the CAP strategies that are in line with the Green Deal’s ambitions. These remote sensing-based controls can be used in the administrative process associated to direct and greening payments compliance. Possible inconsistencies between parcel classifications and farmers declarations should be clarified by in situ checks. In the case of farmers who do not respect greening rules, this may lead paying agencies to impose proportionate sanctions (depending on the scope of the non-compliance and severity) on top of the reduction in greening payments. Therefore, the use of accurate models—and explainable and interpretable—predictions is fundamental in these applications.

The performance of a deep recurrent network was assessed for land use classification from time series of Sentinel-2 data. The overall accuracy reached by the 2-BiLSTM network was 98.7%, outperforming the rest of the classification algorithms evaluated. The obtained accuracy was $\ge$ 91.4% in all cases, which highlights the algorithm robustness, and it excelled reaching to 99.9% over rice crops. Even though the per class accuracy were high, some confusion was also reported mainly over permanent crops.

The best results were achieved using two BiLSTM layers, which indicates that increasing layers is not synonymous to better performance in deep learning approaches. The most relevant information used by the network during training is extracted from the NDVI, B8 (NIR), B4 (red) and $E_{NDVI}$ predictors. From the temporal standpoint the Sentinel-2 images corresponding to the summer period were the most informative. The network’s outputs interpretability assessment exposed the information flow through the network also evidencing the dates in which higher activations of the hidden units were produced.

These analyses help to understand the behaviour of deep learning models in agricultural applications. In particular, in the CAP activities in which payments to farmers must be well-founded, the use of classification models providing explainable predictions are of great interest. The conducted work not only confirm well established knowledge in remote sensing science but also opens the door to new studies in the field of the comprehensibility of deep learning algorithms in agricultural and environmental applications.

## Materials and methods

### Sentinel-2 time series

The European Space Agency (ESA) provides free access to Copernicus Sentinel-2 data from the Sentinels Scientific Data Hub (SSDH). Sentinel-2 mission is composed of two twin satellites (Sentinel-2A and Sentinel-2B) that combined, offer a 5-day period of revisit. Both platforms carry on board the MultiSpectral Imager (MSI) sensor that provides multispectral images in 13 spectral bands covering areas of the visible spectrum, near infrared, and short-wave infrared. The spatial resolution of the data varies depending on the band: the B2 (blue channel), B3 (green channel), B4 (red channel), and B8 (near infrared channel) bands are provided at 10 m; the red-edge bands (B5, B6, B7), the B8a (narrow-near-infrared channel) band, and the short-wave infrared bands (B11 and B12) are provided at 20 m; the B1 (aerosols), B9 (water vapour) and B10 (cirrus) bands are available at 60 m of spatial resolution. The latter is usually used only for atmospheric correction.

**Figure 7:**
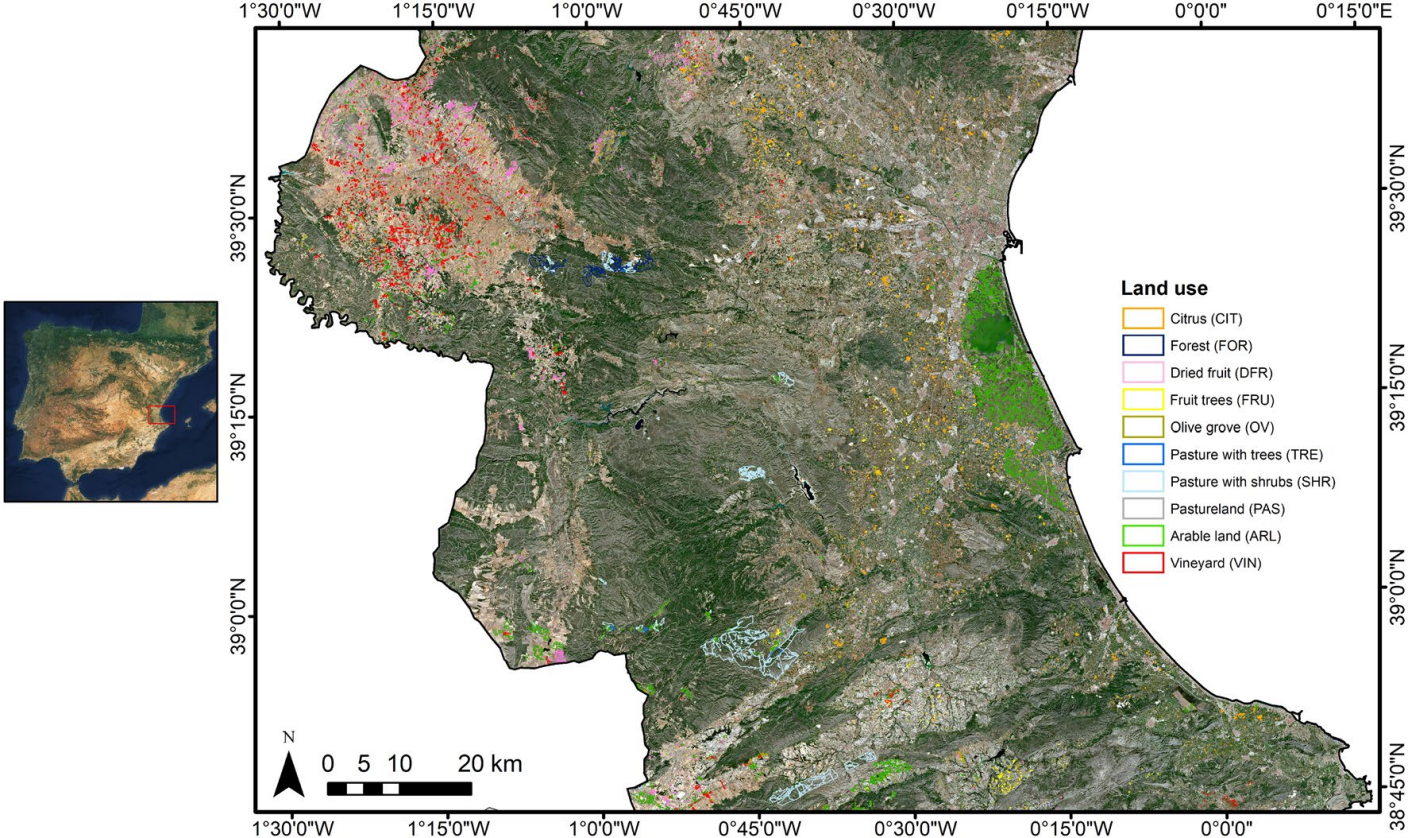
Location of the study area (eastern Spain), and a corresponding Sentinel-2 RGB (B4, B3, B2) composite image acquired on September 13, 2017 with overlaid ground truth. For the sake of visualisation, parcels belonging to rice, fallow, barley, oat, wheat, sunflower, and triticale have been grouped and displayed as arable land (ARL). The maps were generated with the Arcmap v.10.5 software (https://desktop.arcgis.com/es/arcmap/).

Time series of Sentinel-2 data were downloaded from the SSDH covering the 2017/2018 agronomic year from September, 2017 to August, 2018. Since the Valencia province lies on two Sentinel-2 tiles (T30SYJ and T30SXJ), a total of 60 (30 per tile) cloud free images were identified and downloaded over the Valencia province. The Sentinel-2 level 2A product that provides surface reflectance in twelve bands (all except B10) was downloaded. The 20 m and 60 m spatial resolution bands were resampled to 10 m in order to obtain a data set of 10 m in the all twelve bands. Figure 7 shows the location of the study area in Spain, and a 10 m Sentinel-2 RGB (B4, B3, B2) composite image over the area.

In addition to the twelve bands per image, the normalised difference vegetation index^57,58^ (NDVI) computed as $NDVI=\frac{B8-B4}{B8+B4}$, and its entropy (E$_{NDVI}$) were calculated. The use of vegetation indices, and spatial information such as textures, is a common procedure in RS to differentiate classes^50,59,60^. Altogether, time series of 14 features along 30 time steps were used as predictors/inputs in the classification algorithms.

### Ground data

The samples used for training and testing the classification models were provided by the Department of Agriculture, Rural Development, Climate Emergency and Ecology Transition (http://www.agroambient.gva.es/) belonging to the regional government of Valencia in Spain. This information comes from Valencia’s province-wide in situ checks carried out during the 2017/2018 agronomic year. Sixteen land uses were categorised as pasture with trees (TRE), forest (FOR), vineyard (VIN), rice (RIC), pasture with shrubs (SHR), dried fruit (DFR), citrus (CIT), fallow (FAL), barley (BAR), olive grove (OLI), pastureland (PAS), fruit trees (FRU), oat (OAT), wheat (WHE), sunflower (SUN), and triticale (TRI). Table 2 shows the number of samples for every class categorised in the field inspections. The data were geolocated over the Sentinel-2 images to match every sample with its corresponding remote sensing sequence of data. Finally, 70% of the data were used for training the algorithms whereas the remaining 30% were used only for validation.

**Table 2:** Number of pixels identified in the in situ visits. Of those, 70% were used for training and the remaining 30% for validation.

Land use	# samples
TRE	663,995
FOR	495,223
VIN	240,418
RIC	230,935
SHR	165,110
DFR	153,727
CIT	125,161
FAL	84,491
BAR	71,623
OLI	49,829
PAS	33,408
FRU	29,859
OAT	28,754
WHE	11,437
SUN	10,104
TRI	4252
TOTAL	2,398,326

### Bi-directional long short-term memory network (BiLSTM)

LSTM is a special recurrent hidden unit that was proposed to deal with the vanishing gradient problem in RNNs and learn long-term dependencies^23^. Recurrent networks based on LSTM units overcome this drawback by using a gate that controls whether the incoming information is useful or not. Temporal dependencies are taken into account via what is known as the network memory or memory cell. This information flows through each of the network LSTM units, which are composed by three gates: the input ($\mathbf {i}_{t}$), forget ($\mathbf {f}_{t}$), and output ($\mathbf {o}_{t}$) gates. In a time step or instant *t*, the LSTM unit reads the input $\mathbf {x}_{t}$, and the previous hidden state $\mathbf {h}_{t-1}$. Their combination is modulated by an hyperbolic tangent as:

1 $$\begin{aligned} \tilde{\mathbf {c}}_{t}=\tanh (\mathbf {W}_{c}\mathbf {x}_{t}+\mathbf {U}_{c}\mathbf {h}_{t-1} +\mathbf{b}_{c}),\end{aligned}$$

where $\mathbf {W}_{c}$, $\mathbf {U}_{c}$, and $\mathbf{b}_{c}$ are the input weights, the recurrent weights, and the bias, respectively. The input gate determines which information is stored in the memory cell by means of a sigmoid function:

2 $$\begin{aligned} \mathbf {i}_{t}=\sigma (\mathbf {W}_{i}\mathbf {x}_{t}+\mathbf {U}_{i}\mathbf {h}_{t-1}+\mathbf{b}_{i}), \end{aligned}$$

and similarly, the forget gate decides which content of the existing memory cell is forgotten:

3 $$\begin{aligned} \mathbf {f}_{t}=\sigma (\mathbf {W}_{f}\mathbf {x}_{t}+\mathbf {U}_{f}\mathbf {h}_{t-1}+\mathbf{b}_{f}). \end{aligned}$$

The information is updated into the memory cell by adding the information coming from both the input and forget gates, i.e., adding new information from $\mathbf {c}_{t}$, and rules out part of the current memory information:

4 $$\begin{aligned} \mathbf {c}_{t}=\mathbf {i}_{t}\odot \tilde{\mathbf {c}}_{t}+\mathbf {f}_{t}\odot \mathbf {c}_{t-1} \end{aligned}$$

Finally, the output (hidden) state is obtained by the output gate and the updated memory cell as:

5 $$\begin{aligned} \mathbf {h}_{t}=\mathbf {o}_{t}\odot \tanh (\mathbf {c}_{t}), \end{aligned}$$

where the output gate $\mathbf {o}_{t}$ that determines the part of the memory content that will be revealed is given by:

6 $$\begin{aligned} \mathbf {o}_{t}=\sigma (\mathbf {W}_{o}\mathbf {x}_{t}+\mathbf {U}_{o}\mathbf {h}_{t-1}+\mathbf{b}_{o}). \end{aligned}$$

**Figure 8:**
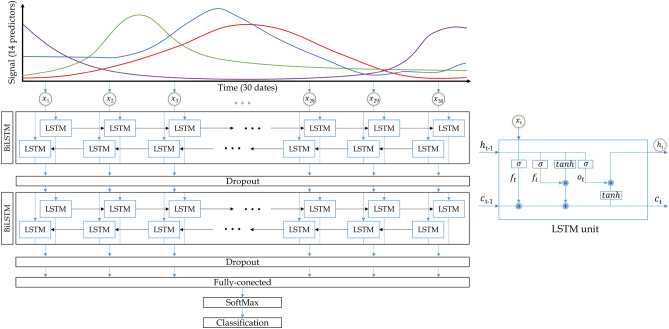
Architecture of the 2-BiLSMT network (left), and LSTM unit components (right). In our case, 14 features along 30 time steps were used as predictor variables.

LSMT units can be combined to obtain a bi-directional long short-term memory (BiLSTM) network. The BiLSTM networks are formed by two LSTM units per time step, and take into account not only past temporal dependences but also information of future time states. Hence, BiLSTM networks learn from the complete time series at each time step thus having a global view of the sequences^61^. In this work an deep network architecture composed by a combination of two BiLSTM layers (2-BiLSTM) was used, as shown in Fig. 8. The main components of the 2-BiLSTM network are: (1) the input layer formed by the 14 Sentinel-2 selected time series, (2) two BiLSTM layers with 100 hidden units followed by a 50% dropout layer to avoid overfitting, (3) a fully-connected layer connecting the units to every activation unit of the next layer, (4) a softmax layer that computes the probability of every class in the network output, and (5) the output layer containing the predictions.

### Evaluation and interpretability

The accuracy of the proposed 2-BiLSTM network was assessed by computing the overall accuracy in a first step. The obtained accuracy was compared with the ones obtained using other network architectures, namely three similar networks formed by a single (BiLSTM), three (3-BiLSTM), and four BiLSTM layers (4-BiLSTM), as well as with different machine learning classification algorithms: decision trees (DT), k-nearest neighbours (k-NN), neural networks, support vector machine (SVM), and random forests (RF).

The 2-BiLSMT network behaviour was subsequently addressed by identifying the most relevant inputs (i.e., predictors) in the spectral and temporal domains. This was achieved by means of an added-noise permutation approach consisting in the adding of Gaussian white noise $\mathscr {N}(0,\,\sigma ^{2})$, being $\sigma ^{2}$ the 3% of the perturbed signal amplitude. The noise was added to a single predictor in all time steps remaining the rest of the predictors unperturbed. This process was repeated for every predictor, thereby obtaining different accuracies for every case. The relevance of each predictor was computed as the difference between the accuracy obtained with no perturbation and the obtained when the perturbation was applied. The results were normalised with respect to the most relevant predictor. This approach was carried out again to identify the most relevant date. In this case the perturbation was added to all predictors in a single time step leaving the rest of the dates unperturbed.

The interpretability of the classifications was addressed by showing how predictions and their probability change between time steps. In addition, the BiLSTM hidden units activation was visualised, and the average squared activations in the temporal domain were computed to analyse how the information flows through the network.

## Supplementary information

Supplementary Information
