# Supplementary figures and images for: Understanding deep learning in land use classification based on Sentinel-2 time series
Source: Sci Rep. 2020 Oct 14;10:17188. doi: 10.1038/s41598-020-74215-5 (PMC7560821; doi:10.1038/s41598-020-74215-5)

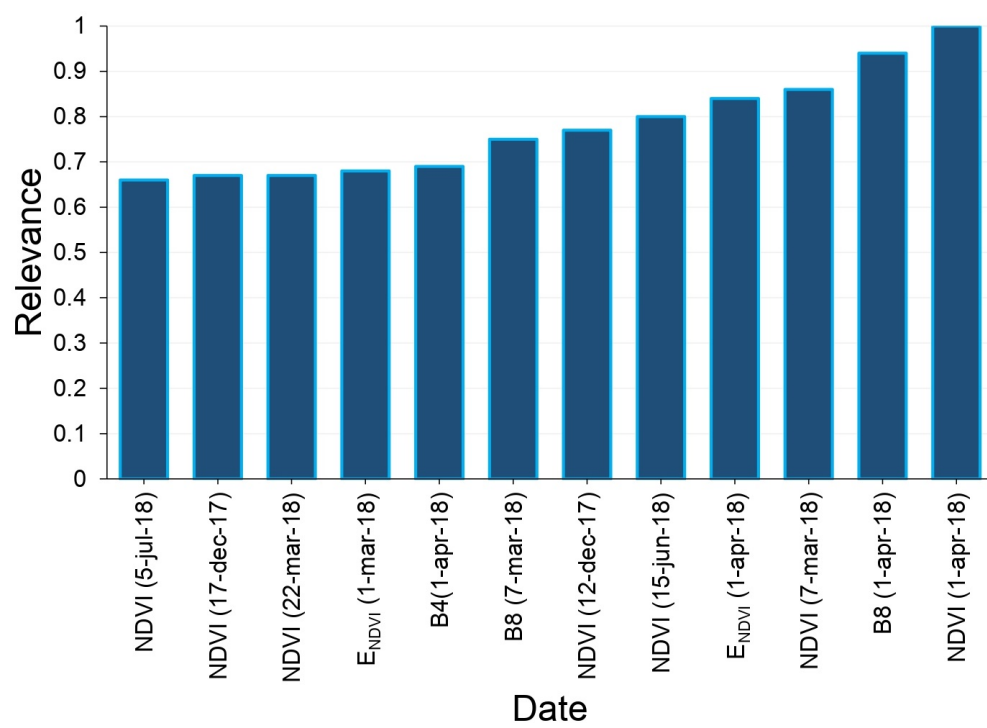

**Figure S2.** Relevance of the top ten predictors provided by the RF classification algorithm.

Supplement: Supplementary file 1 — Supplementary Information [file 41598_2020_74215_MOESM1_ESM.pdf]
